# Antineoplastic multi-drug chemotherapy to sensitize tumors triggers multi-drug resistance and inhibits efficiency of maintenance treatment inglioblastoma cells

**DOI:** 10.17179/excli2022-5556

**Published:** 2023-01-04

**Authors:** Oguzhan Doganlar, Zeynep Banu Doganlar, Suat Erdogan, Emre Delen

**Affiliations:** 1 Faculty of Medicine, Department of Medical Biology, Trakya University, Edirne, Turkiye; 2 Faculty of Medicine, Department of Neurosurgery, Trakya University, Edirne, Turkiye

**Keywords:** glioblastoma, multi-drug resistance, apoptosis, autophagy, angiogenesis, U87 MG

## Abstract

Combinations of the well-known antineoplastic agents 5-fluorouracil (5-Fu), cisplatin, and paclitaxel are employed to increase radiotherapy/immunotherapy efficacy against persistent and resistant tumors. However, data remains needed on the hormetic, chronic, and long-term side effects of these aggressive combination chemotherapies. Here we investigated cellular and molecular responses associated with these combined agents, and their potential to induce multi-drug resistance against the temozolomide (TMZ) and etoposide (EP) used in glioblastoma maintenance treatment. We analyzed resistance and survival signals in U87 MG cells using molecular probes, fluorescent staining, qRT-PCR, and immunoblot. Repeated treatment with combined 5-Fu, cisplatin, and paclitaxel induced cross-resistance against TMZ and EP. Resistant cells exhibited elevated gene/protein expression of MRP1/ABCC1, ABCC2, BRCP/ABCG2, and GST. Moreover, they managed oxidative stress, cell cycle, apoptosis, and autophagy signaling to ensure survival. In these groups TMZ and etoposide efficiency dramatically reduced. Our result suggests that combined high-dose treatments of classical antineoplastic agents to sensitize tumors may trigger multi-drug resistance and inhibit maintenance treatment. When deciding on antineoplastic combination therapy for persistent/resistant glioblastoma, we recommend analyzing the long-term hormetic and chronic effects on cross-resistance and multi-drug resistance in primary cell cultures from patients.

See also the Graphical Abstract(Fig. 1).

## Introduction

Multi-drug resistance (MDR) is a strong resistance mechanism of cancer cells characterized by a cross-resistance phenotype; it acts against chemically distinct drugs and targets multiple different signaling pathways (Szakács et al., 2006[39]; Wang et al., 2017[43]). The MDR phenotype is generally activated by ATP-binding cassette (ABC) membrane transporters (specifically multi-drug resistance proteins, MRPs, and breast cancer resistance protein, BCRP) and other types of pump mechanisms such as glutathione S-transferases (GSTs) (Gottesman et al., 2002[17]; Wu et a., 2014[44]; Bao et al., 2020[6]). Typically, high-dose combined drug administration is required to establish clinically effective chemotherapy in cancer cells with the MDR phenotype. However, such a treatment is often impossible to manage and culminates in a high incidence of mortality, especially when cross-resistance develops. As MDR is a multi-factorial process, it is essential to clearly determine the molecular mechanisms of resistance in order to establish a manageable chemotherapy treatment model.

Glioblastoma multiforme (GBM) accounts for around 70 % of all tumors in the central nervous system. Its highly aggressive characteristics, including rapid proliferation, metastatic potential, high heterogeneity, infiltrative nature, and a propensity for recurrence, lead to a very poor prognosis; the median survival period is 15 months, and only 27 % of patients reach two years' survival even if given combined treatment involving surgical resection, radiotherapy, and high-dose chemotherapy (Stupp et al., 2014[38]; Morgan, 2015[26]). Furthermore, GBM cells exhibit the MDR phenotype and are inherently resistant to a wide range of chemotherapy agents (Da Ros et al., 2018[12]). Indeed, a number of studies support MDR as one of the most important problems in GBM treatment (Zanders et al., 2019[46]). Due to MDR, the overwhelming majority of GBM patients cannot be fully cured and must receive maintenance treatment throughout the remainder of their lives. 

Temozolomide (TMZ) is at present the primary maintenance therapy choice for GBM, being used after the main therapy to prevent tumor growth; etoposide (EP) is rarely used (Oshiro et al., 2009[29]). However, recent research has shown that more than 60 % of glioblastoma patients treated with TMZ develop resistance, and 15-20 % of patients do not benefit from TMZ despite not having received it previously (Zanders et al., 2019[46]). Although many studies have been published on the causes and molecular mechanisms of TMZ resistance in GBM patients treated with long-term TMZ (Jiaper et al., 2018[21]), the etiologic and mechanistic features of strong drug resistance and treatment failure in those never exposed to TMZ remain unclear.

Recently, double and triple combinations of well-known antineoplastic agents 5-fluorouracil (5-Fu), cisplatin, and paclitaxel have begun to be used to increase the efficacy of radiotherapy or immunotherapy drugs in treating persistent and resistant tumors such as GBM (Jeyapalan et al., 2014[20]; Zanders et al., 2019[46]), head and neck cancers (Adkins et al., 2018[2]; Tsakonas et al., 2020[41]), esophageal cancer (Steber et al., 2021[35]), and gastric cancer (Xu et al., 2019[45]). While no single one of these drugs showed clinical efficacy, their combined application for periods of 1-3 months has provided promising results. However, there is not yet sufficient data on the hormetic, chronic, and long-term side effects of these aggressive combination therapies. Notably, single application of 5-Fu (Oguri et al., 2007[28]; Kurasaka et al., 2021[23]), cisplatin (Aldossary, 2019[4]; Makovec, 2019[25]), and paclitaxel (Podolski-Renić et al., 2011[31]; Aldonza et al., 2017[3]) to cancer cells has been shown to affect many molecular signaling pathways, especially detoxification mechanisms, leading to the development of cross- or multi-drug resistance. Moreover, their combined use synergistically increases drug resistance (Patel et al., 2021[30]). In addition, GBM tumors in particular pose many obstacles to the functionality of chemotherapy agents, such as overcoming the blood-brain barrier, the wide variety of cells in the tumor microenvironment, and the tumor's infiltrative properties. Consequently, some tumor cells will be able to escape lethal doses of the chemotherapy agents, but will also be exposed to sub-lethal doses.

Our hypothesis is that cancer cells exposed to long-term and sub-lethal levels of combination chemotherapy may develop drug resistance, with the resistant population subsequently increasing via a selection effect. Moreover, use of multiple drugs can activate many resistance mechanisms simultaneously, thus cancer cells not having previously encountered TMZ or EP can become resistant to these maintenance therapies via developing multi-drug resistance. 

Here, we test our hypothesis by subjecting U87 MG GBM cells to long-term combined 5-Fu, cisplatin, and paclitaxel at sub-lethal concentrations. After 15 passages (generations), we assessed the responses of normal and resistant populations to TMZ and EP treatment and characterized expression of important genes and proteins in cellular signaling pathways known to be associated with multi-drug resistance in GBM.

## Materials and Methods

### GBM cells and culture conditions

The U87 MG (ATCC® HTB-14™) human glioblastoma cell line was obtained from the American Type Culture Collection (ATCC) and was grown in Eagle's supplemented minimum essential medium (EMEM; Lot No. 2062257), further supplemented with heat-inactivated 10 % fetal bovine serum, 1 mM L-glutamine (Gibco Life Technologies), 100 U/mL penicillin, and 100 μg/mL streptomycin (Invitrogen). Cells were cultured in a humidified incubator at 37 °C and 5 % CO_2_.

### The combined 5-fluorouracil (5-Fu), cisplatin (CP), and paclitaxel (PX) treatment and in vitro toxicity model

A multi-drug mixture was prepared with 5-fluorouracil (5-Fu) (CAS 51-21-8, F6627, Sigma Aldrich, USA), cisplatin (CP) (CAS 15663-27-1; sc-200896, Santa Cruz, USA), and paclitaxel (PX) (CAS 33069-62-4, T7191, Sigma Aldrich, USA), which have different modes of action and are extensively used for tumor sensitization. In the first step, we determined sub-lethal doses of each chemotherapeutic by testing a range of concentrations: 1.0-1000 µM for 5-Fu, 0.78-200 µM for CP, and 0.5-500 nM for PX. We calculated the IC_50_ and IC_20_ doses of these drugs by probit analysis using data obtained from MTT [3-(4,5-dimethylthiazol-2-yl)-2,5-diphenyltetrazolium bromide] assays (Table 1(Tab. 1)). To create the multi-drug therapy, we used the IC_20_ values obtained over 48 hours for 5-Fu and CP and 72 hours for PX; this combination was applied to U87 cells and yielded cell viability of 64.67±14.04 % after 72 hours of incubation. We concluded this combination dosage appropriate for establishing an *in vitro* toxicity model.

**Figure 2:**
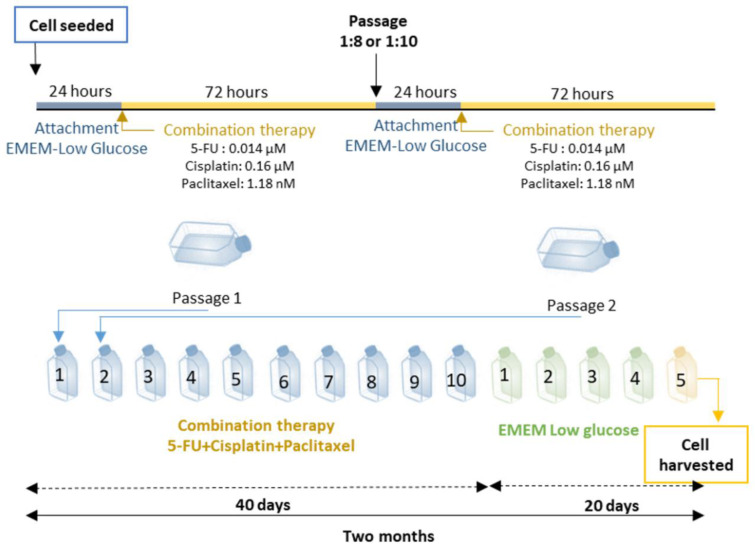
Combined therapy (5-fluorouracil, cisplatin and paclitaxel) schedule to establish drug resistance. After plating U87 cells, three drugs at doses of IC_20_ were added to EMEM low glucose medium for 72 h. Combination treatment lasted for 40 days and 10 passages, after which the cells were grown for 5 more passages in EMEM low glucose medium without any drugs to test whether heritable resistance was formed. The cells were then exposed to chemotherapy agents to model maintenance treatment (Etoposide and TMZ) and MTT assays were performed.

The chemotherapy agents were dissolved in DMSO (dimethyl sulfoxide), prepared fresh for each treatment, then diluted with molecular grade water to a final DMSO concentration of 0.5 % and used in combined application. The multi-drug mixture was initially applied to 75 cm^2^ cell culture flasks. After every 72 hours, the cells were passaged to ensure cell proliferation and then given 24 hours to reach confluence and adhere, after which drug administration was repeated. In total, ten passages and combined treatment cycles were carried out, paralleled by control cells that received an equal concentration of vehicle. After completing the ten passages (which took approximately 40 days), the cells were then maintained for five more passages in complete EMEM medium without any drug application so as to eliminate any temporary response. Cells were harvested at the 15^th^ passage and the MTT test was performed with etoposide (EP) and temozolomide (TMZ); these cells were used in all other experimental assays (Figure 2(Fig. 2)).

### Cell viability assay (MTT test)

U87 MG cells were seeded into 96-well plates (3000 cells/per well) and incubated overnight under standard cell culture conditions (see above). The cells were then treated with selected concentrations of drugs at a final vehicle concentration of 0.5 % DMSO (Merck, USA). After incubation for 24, 48, or 72 h, the cells were treated with (3-4,5-dimethylthiazol-2-yl)-2,5-diphenyltetrazolium bromide (MTT, 5 mg/mL; Thermo Fisher Scientific, USA) for 4 h. Next, the cell medium was replaced with DMSO (102950 Supelco, USA) and the absorbance was read at both 492 and 570 nm using a reference wavelength of 650 nm (Thermo Multiskan GO, USA). IC_50_ and IC_20_ values were calculated by probit analysis using SPSS 20 software.

### Detection of apoptotic and necrotic cells and live cell imaging

Multidrug-treated U87-R or untreated U87 cells (approx. 15000 per well) were seeded onto 24-well plates. After 18 h, the respective IC_50_ doses of EP and TMZ were each administered for 6 and 24 hours. Percentages of apoptotic, necrotic, and live cells were determined by a Tali® image-based cytometer using the Tali® Apoptosis Kit (Life Technologies, USA); additionally, live-cell imaging was performed and the results analyzed according to a protocol described previously (Doğanlar et al., 2020[15], 2021[16]) using the NucBlue® Live Ready Probes® Reagent, CM-H_2_DCFDA dye (Life Technologies, USA), and acridine orange-ethidium bromide (AO/EB) (Sigma Aldrich, USA). 

### Wound-healing assay

Multidrug-treated U87-R or untreated U87 cells were seeded in 25-ml tissue culture flasks and allowed to grow until reaching 85-90 % confluence. The adhered cells were then scratched with a sterile 200-μL pipette tip and the debris washed away with EMEM. Next, we added 1 mL of serum-free EMEM and exposed the cells to IC_50_ concentrations of EP and TMZ (or vehicle). Wound closure was determined according to the protocol established by Doğanlar and colleagues (2020[15]).

### Total RNA isolation, reverse transcription, and real-time PCR (qRT-PCR) assay

Total cellular RNA was extracted using a PureLink™ RNA Mini Kit (Thermo Scientific, USA) according to the manufacturer's protocol. The cDNA libraries were prepared using the High-Capacity cDNA Reverse Transcription Kit (Thermo Scientific, USA), and qRT-PCR was performed using Power SYBR™ Green PCR Master Mix (Life Technologies, USA) on a Quant Studio 5 real-time PCR machine (Life Technologies, USA) to determine relative fold changes in target gene expression. Primers and PCR conditions are given in Table 2(Tab. 2). Fold change was calculated using the 2^-ΔΔCt^ method, with *β-actin* and *GAPDH* used as multiple internal controls.

### Immunoblot assay

Total protein was extracted using the RIPA Lysis Buffer System (sc-24948, Santa Cruz, USA) according to the kit protocol. Protein levels were determined by Western blot assay using the NuPAGE™ 4 to 12 %, Bis-Tris, 1.0 mm Mini Protein Gel (NP0321) with the WesternBreeze™ Chemiluminescent Kit (ThermoFisher Scientific, USA) according to the method of Doganlar and colleagues (2021[16]). Seven primary antibodies were utilized: GST tag antibody (GST 3-4C), ABCC1 (NB400-156), ABCC2 antibody (NBP1-69023), ABCG2 antibody (BXP-21), LC3B antibody (NB100-2220) (Novus Biological, USA), and actin antibody (ACTN05 (C4)) (ThermoFisher Scientific). Protein expression levels were normalized to β-actin, and band intensity was measured and calculated as per our previous study (Doğanlar et al., 2021[14]).

### Statistical analysis

Differences between multidrug-treated U87-R and untreated U87 groups in percentages of live, dead, and apoptotic cells, cell viability, and relative fold change in gene expression were assessed by one-way analysis of variance (ANOVA) with the Tukey HSD test or Tamhane's T2 as appropriate for the normalized test results. Additionally, the Mann-Whitney U test or independent samples *t*-test was used in two-group comparisons. Results having *p*-values of less than 0.05 were considered significant. We analyzed and plotted our results using SPSS 20 and GraphPad Prism 7.

## Results

### Development and characterization of the MDR phenotype in GBM cells

**Figure 3:**
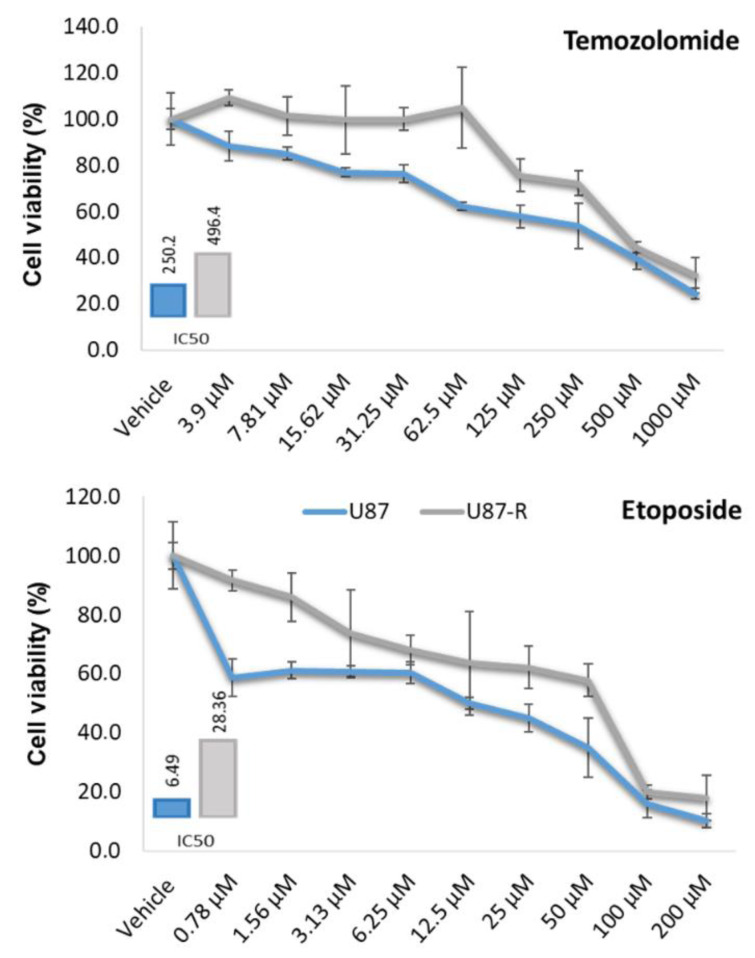
Figure 3. Cell viability (%) in resistant U87-R and normal U87 cell populations treated with nine different doses of temozolomide and etoposide for 72 h. MTT data were used to calculate IC_50_ values (µM) using the probit analysis function of SPSS20. All percentages are given as mean ± SD, n = 6.

To determine whether glioblastoma cells given prolonged exposure to sub-lethal doses of 5-Fu, cisplatin, and paclitaxel develop multi-drug resistance, we first tested the cells with drugs having structures and modes of action distinctly different from those agents. Specifically, after five additional passages without drugs to eliminate spontaneous resistance, we treated the U87 cells with increasing concentrations of EP or TMZ for 72 h and determined cell proliferation by MTT test (Figure 3(Fig. 3)). Cells subjected to the sub-lethal chemotherapeutic mix showed strong resistance to EP and TMZ; at the 72-hour mark, U87-R cells exhibited respective IC_50_ values for EP and TMP that were approximately 4.36- and 1.98-fold greater than those of control cells. Our results thus demonstrate that repeated application of combined chemotherapy to U87 cells induces development of resistance to both of the unrelated drugs EP and TMZ.

**Figure 4:**
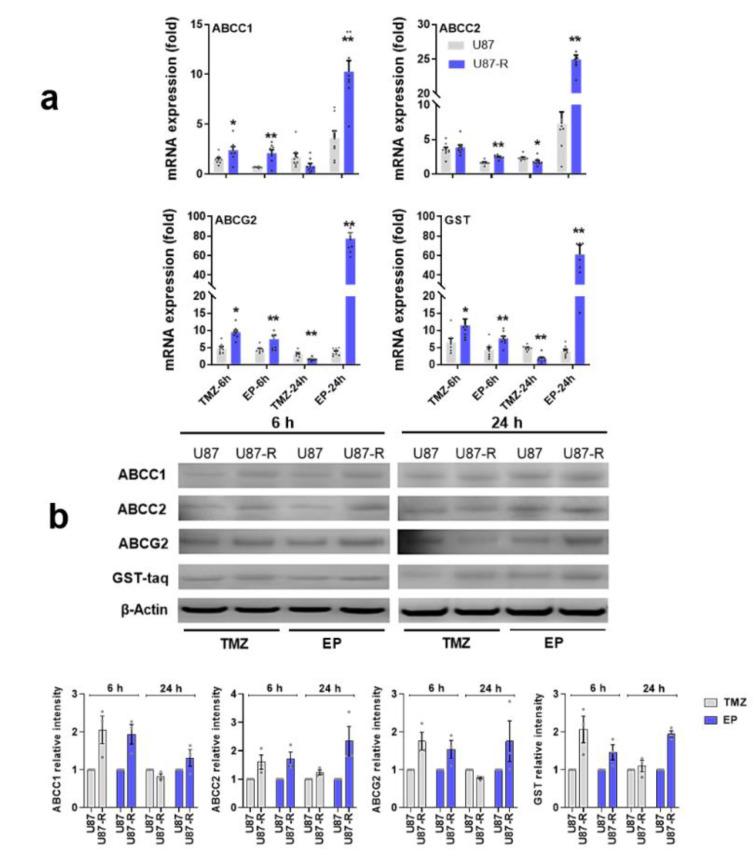
a) Relative fold change of *ABCC1*, *ABCC2*, *ABCG2*, and *GST* in temozolomide (TMZ)- and etoposide (EP)-treated resistant U87-R and non-resistant U87 cells as determined by quantitative real-time PCR. All data were normalized to β-actin and GAPDH are given relative to control (control=1, not shown in figure). Data are represented as mean ± SE, n=8. *indicates significantly different values in U87-R vs U87 groups, Mann-Whitney U test: *p ≤0.05; ** *p* ≤0.01. b) Western blot analysis of ABCC1, ABCC2, ABCG2, and GST proteins (relative density normalized to β-actin). Data are represented as mean ± SE, n=3. Control: vehicle-treated control; TMZ: temozolomide, IC_50_: 250.2 μM; EP: etoposide, IC_50_: 6.49 μM

In the second step, we focused on determining the mechanism of this resistance in the U87-R population. The main detoxification mechanisms and efflux pumps involved in MDR are ATP-binding cassette membrane transporters (ABC transporters) and glutathione S-transferase. To assess whether the observed EP and TMZ resistance originates from an MDR mechanism, we treated U87-R cells with the IC_50_ value of EP or TMZ, and investigated at 6 and 24 h the molecular activation of MRP1/ABCC1, ABCC2, BRCP/ ABCG2, and GST (Figure 4(Fig. 4)). 

Our results indicate that both EP and TMZ significantly activate MDR markers in a time-dependent manner. In resistant U87-R cells, ABC transporters and *GST* alike had significantly increased gene expression compared to normal U87 cells after six hours of drug administration (except *ABCC2* for TMZ treatment). After 24-hour treatment, however, MDR markers were reduced in TMZ-treated U87-R cells compared to controls, while gene expression levels increased significantly in all groups treated with EP. Western blot analysis supported our gene expression results; the highest ABC transporter protein levels after TMZ treatment were observed in the U87-R group at six hours, while EP treatment resulted in especially evident increases after 24 hours. 

### Lower oxidative stress, higher cell viability, and weak apoptosis signal in TMZ- or EP-treated resistant U87-R populations

**Figure 5:**
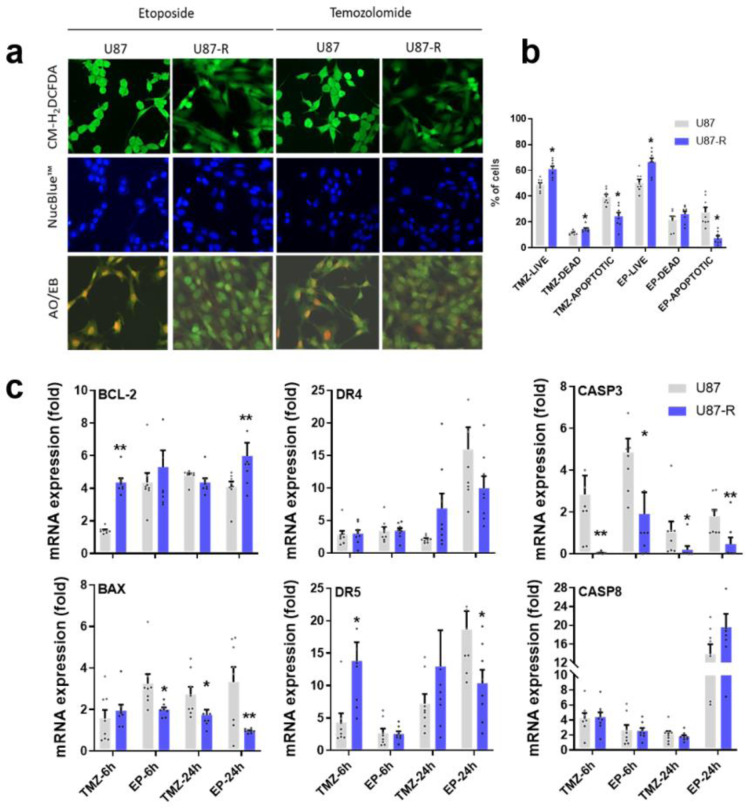
a) Detection of oxidative stress levels and nuclear apoptotic bodies in resistant U87-R and non-resistant U87 cells after treatment with etoposide (EP) and temozolomide (TMZ) at IC_50_ doses; cells were labeled with CM-H_2_DCFDA and stained with acridine orange-ethidium bromide (AO/EB) and Nuc-blue. b) Percentages of live, dead, and apoptotic cells among U87-R and U87 cells treated with EP and TMZ at IC_50_ doses; cells were stained with annexin V/propidium iodide (PI) and states tallied using a Tali image-based cytometer. All percentages are given as mean ± SE, n = 8. *indicates significantly different values compared to respective vehicle-treated control, determined by one-way ANOVA and the Tukey HSD test (*p* ≤ 0.05). c) Relative fold changes of *BCL-2*, *BAX*, *DR4*, *DR5*, caspase-3, and caspase-8 genes in EP- and TMZ-treated U87-R and U87 cells as determined by quantitative real-time PCR. All data were normalized to β-actin and GAPDH expression and are given relative to control (control=1, not shown in figure). Data are presented as mean ± SE, n = 8. *indicates significantly different values in U87-R vs U87 groups, Mann-Whitney U test: *p ≤0.05; ** *p* ≤0.01; Control: vehicle-treated control; TMZ: temozolomide, IC_50_: 250.2 μM; EP: etoposide, IC_50_: 6.49 μM. U87 cells.

Oxidative stress, cell cycle arrest, and deterioration in nuclear morphology are major cellular events that initiate apoptosis following treatment with TMZ or EP. To qualitatively assess the efficiency of TMZ and EP treatment on U87 and U87-R populations, we stained live cells with CM-H_2_DCFDA (for oxidative stress), Nucblue, and the dual stain AO/EB (for apoptosis). In the U87 population, both TMZ and EP treatment induced strong oxidative stress, caused apoptotic body formation, and triggered the apoptosis response. In U87-R cells, however, oxidative stress, apoptotic body formation, and especially early and late apoptotic cells were all significantly reduced (Figure 5a(Fig. 5)). Next, we quantified these findings via determining the percentages of live, necrotic, and apoptotic cells using Tali image-based cytometry with Annexin V:propidium iodide (PI) staining. Following TMZ and EP treatment, we observed a 13.4-15.7 % increase in non-stained live cells among U87-R cells compared to U87 cells, along with similar reductions of 15.05-17.91 % in Annexin V-positive (apoptotic) and Annexin V:PI-positive (late apoptotic) cells, confirming reduction of early and late apoptosis in the resistant U87-R population. In the same experimental conditions, the two populations did not differ in rate of necrotic death (characterized by PI-positive cells), but necrotic responses to EP and TMZ differed, with 1.4-1.6 times higher necrotic death being observed with EP treatment compared to TMZ treatment (Figure 5b(Fig. 5)). 

To further demonstrate inhibition of apoptosis in the resistant population, U87 and U87-R cells were treated with IC_50_ doses of TMZ and EP for 6 and 24 h. As shown in Figure 5c(Fig. 5), treatment of U87-R cells with either drug resulted in induction of *BCL2* and significant decrease of the *BAX*/*BCL2* ratio. In agreement with this initial anti-apoptotic signal, the U87-R cell population exhibited significantly decreased caspase-3 gene expression, indicating a weak mitochondrial apoptosis signal. To investigate the effects of TMZ and EP treatment on the extrinsic apoptosis pathway in U87 and U87-R populations, we also measured relative gene expression of caspase-8 and death receptor 4 (*DR4*) and 5 (*DR5*), which are the main players in that pathway. While *DR4* expression was not significantly different between U87 and U87-R cells, *DR5* increased significantly at six hours after TMZ treatment and 24 hours after EP treatment in U87-R cells relative to U87 cells. Meanwhile, strong caspase-8 expression was observed in both groups, especially after EP treatment, with increases of 13.1-fold in U87 cells and 21.4-fold in U87-R cells. However, Tali image-based cytometer results indicated a significantly reduced proportion of apoptotic U87-R cells, while the necrotic cell population was increased (Figure 5a(Fig. 5)). These data suggest that in the context of EP treatment, caspase-8 may promote necrosis induced by the TNF-α/caspase-8 axis in both U87 and U87-R cells. Taken together, these findings support that the mitochondrial (intrinsic) apoptosis pathway is significantly less active in the resistant U87-R cell population compared to U87 cells given the same IC_50_ doses of TMZ and EP.

### Autophagy signaling in TMZ- and EP-treated normal U87 and resistant U87-R cells 

**Figure 6:**
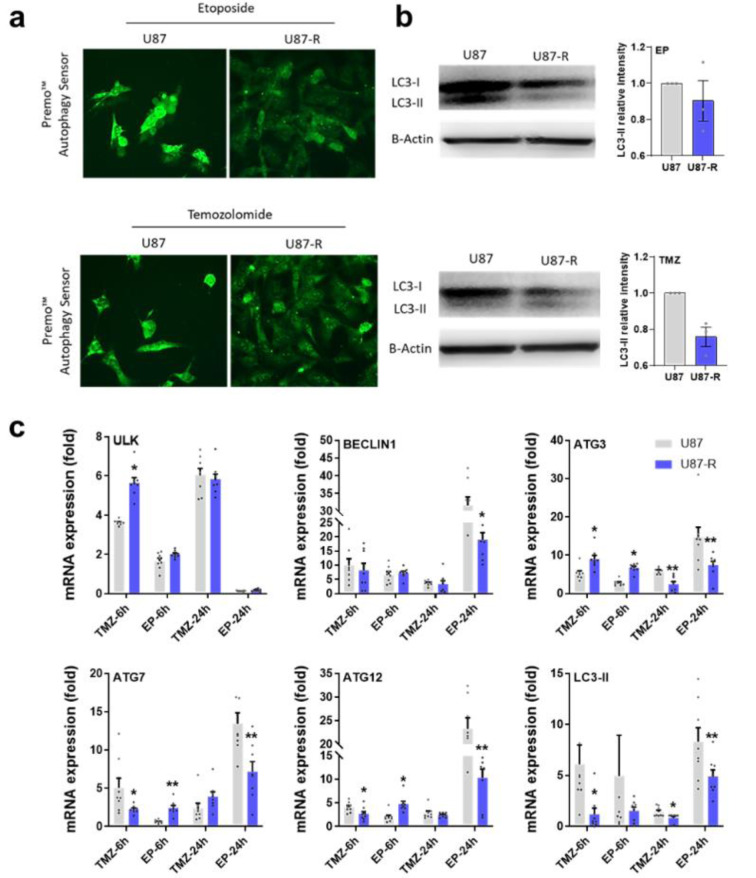
a) Detection of autophagosome formation labeled with Premo autophagy sensor in resistant U87-R and non-resistant U87 cells after treatment with etoposide (EP) and temozolomide (TMZ) at IC_50_ doses, b) Western blot figures with relative intensity of LC3-II protein (relative intensity normalized with β-actin). Data are presented as mean ± SE, n=3. c) Relative fold change determined by quantitative real-time PCR (qRT-PCR) analysis of ULK, BECLIN1, ATG3, ATG7, ATG12 and LC3-II genes in EP and TMZ-treated resistant U87-R and non-resistant U87 cells. All data were normalized with β-actin expression and are given as relative to control (control=1 not shown in figure). Data are presented as mean ± SE, n=8*indicates significantly different values in U87-R vs U87 groups, Mann Whitney U test: *P ≤0.05; ** P ≤0.01; Control: Vehicle-treated control, TMZ: temozolomide IC_50_: 250.2 μM, etoposide IC_50_: 6.49 μM

To assess the autophagy signal induced by IC_50_ doses of TMZ and EP in U87 or U87-R brain tumor cell lines, we investigated expression of autophagy pathway genes in the ULK/BECLIN1, ATG12/ATG7, and ATG3/ LC-II/autophagasome axes. We also determined regulation of the autophagy biomarker LC3-I/LC3-II using Premo Autophagy Sensor molecular probe staining and Western blot assay. According to our results, TMZ and EP treatments in U87 cells significantly decreased cell population and induced strong autophagy signals, the latter also supported by increased LC3-II protein level. However, the resistant U87-R cells clearly exhibited dense cell population and less autophagosome formation, with Western blot analysis revealing a lower level of LC3-II protein (Figure 6a(Fig. 6)). Compared to the U87 group, the resistant U87-R population also demonstrated significant increase in *ULK* gene expression after six hours of TMZ treatment, while *BECLIN1* decreased significantly after 24 hours of EP application. Meanwhile, while resistant cells exhibited increased *ATG7* and *ATG12* gene expression upon short-term administration of chemotherapy agents, levels were lower than in U87 cells, especially after 24 hours. A similar situation was seen for *ATG3* and also for *LC3-II*, which encodes the key molecule in the ATG3/LC3-II axis (Figure 6b(Fig. 6)).

### Angiogenesis signaling in TMZ- and EP-treated normal U87 and resistant U87-R cells 

**Figure 7:**
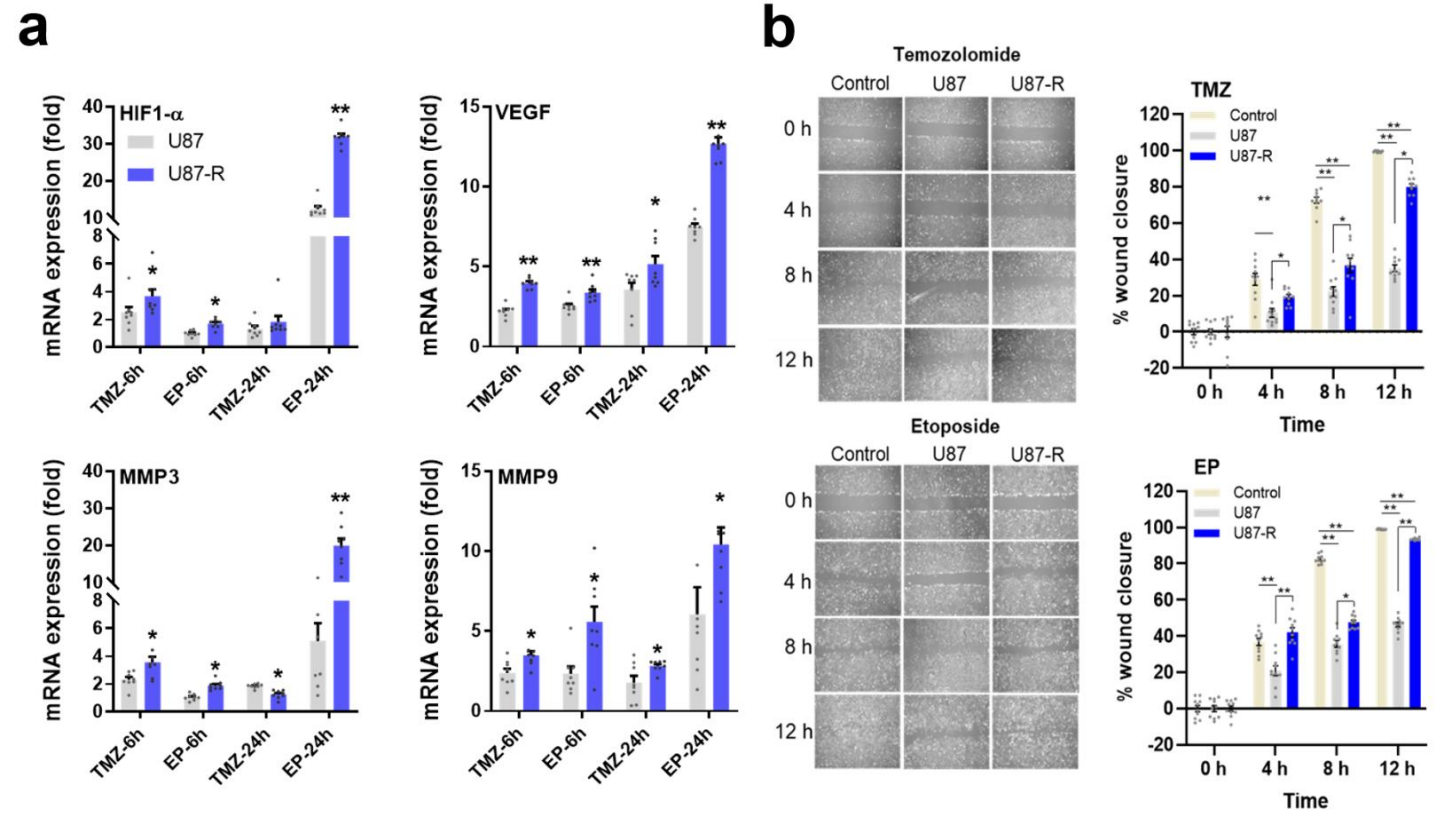
a) Relative fold change of *VEGF*, *HIF1-α*, *MMP3*, and *MMP9* in EP- and TMZ-treated U87-R and U87 cells as determined by quantitative real-time PCR. All data were normalized to β-actin and GAPDH and are given relative to control (control=1, not shown in figure). Data are presented as mean ± SE. n=8 *indicates significantly different values in U87-R vs U87 groups, Mann-Whitney U test: *p ≤0.05; ** *p* ≤0.01 b) Cell migration and wound closure as determined by wound healing assay (for 2, 4, 8, and 12 h) in TMZ- or EP-treated resistant U87-R and non-resistant U87 cells. Data are presented as mean ± SE. n=10 *indicates significantly different values in U87-R vs U87 groups, oneway-ANOVA, Tukey HSD test *p ≤0.05; ** p ≤0.01; Control: Vehicle-treated control, TMZ: temozolomide IC_50_: 250.2 μM, etoposide IC_50_: 6.49 μM.

To investigate the MDR-associated invasiveness of GBM using our drug-treated chemo-resistant cell model, we first measured relative expression of genes in the HIF1-α/VEGF/MMPs axis, which are linked to hypoxia and angiogenesis (Figure 7a(Fig. 7)). We found expression of this axis to be significantly higher in the resistant U87-R population compared to normal U87 cells. Overall, the highest expression values were found after 24-hour treatment with EP, with resistant cells exhibiting respective increases of 12.8-fold, 32.4-fold, 9.3-fold, and 19.6-fold for *VEGF*, *HIF1-α*, *MMP3*, and *MMP9*. Furthermore, wound healing assays revealed the U87-R population to have considerably higher rates of both cell migration and wound closure than U87 cells under the same treatment conditions (Figure 7b(Fig. 7)).

See also the Supplementary data.

## Discussion

In this study, we showed that repeated treatment of the U87 cell line with IC_20_ doses of 5-Fu, cisplatin, and paclitaxel caused statistically significant and irreversible drug resistance. Moreover, these drug-resistant cells also showed resistance to TMZ and EP, which have different chemical structures and mechanisms of action. Appropriately, the resistant cells exhibited significant overexpression of genes involved in multi-drug resistance (*MRP1/ABCC1*, *MRP2/ABCC2*, and *BRCP/ABCG2*) and detoxification (*GST*). The ABCC1 protein features in resistance to taxane group drugs, and many studies have shown that overexpression of ABCC group proteins is a marker of resistance to drugs such as anthracyclines, vinca alkaloids, epipodophyllotoxins, cisplatin, etoposide, and epirubicin (Borst et al., 2000[7]; Toyoda et al., 2008[40]; Choi et al., 2011[9]; Zhang et al., 2012[47]). BRCP/ABCG2 is reportedly responsible for multi-drug resistance due to pumping drugs out of cancer cells, and GST also contributes via the detoxification and deactivation of anticancer drugs (Arévalo et al., 2017[5]; Doğanlar et al., 2020[15]). Our results are consistent with these previous findings and we deduce that ABCC1, ABCC2, ABCG2, and GST contribute to drug resistance mechanisms in U87-R cells. 

Regarding the determination of apoptosis, we investigated the expression of genes in both extrinsic and intrinsic apoptosis pathways. Resistant cells exhibited higher expression of an anti-apoptotic gene (*BCL2*) and lower expression of an apoptotic gene (*BAX*) than did normal cells. Additionally, after 24 hours of EP treatment, expression of the extrinsic apoptotic pathway receptors *DR4* and *DR5* was significantly reduced in U87-R cells relative to normal cells. Meanwhile, statistically significant activation of caspase-8 was observed after 24 hours of EP application in both cell types, but levels were significantly lower in resistant cells relative to normal cells. Upon consideration of both fluorescent staining results and Tali analyses, we concluded that this caspase-8 activation was not directly related to the apoptosis pathway, but instead was associated with the necrotic death pathway, particularly through the TNF-α/caspase-8 axis. Activation of this pathway might explain the high degree of necrotic death seen in cytometry analysis after EP treatment. Similar to our findings, upregulation of antiapoptotic BCL2 and downregulation of BAX were reported in recurrent glioblastoma (Strik et al., 1999[37]). Additionally, dysregulation of apoptosis-related genes in primary tumors causes apoptosis resistance and decreased BAX expression, which are associated with poor prognosis (Ruano et al., 2008[34]), and overexpression of anti-apoptotic proteins causes inhibition of caspase expression (Stegh et al., 2008[36]). Our results thus parallel the literature, with upregulation of *BCL2* and downregulation of *BAX* and caspase-3 indicating apoptosis resistance in U87-R cells. 

Gliomas are reportedly more resistant to apoptosis-inducing treatments than to autophagy-inducing treatments. Autophagic processes are induced by disturbance of a pathway controlled by rapamycin (Iwamaru et al., 2007[18]), with ULK-induced phosphorylation of BECLIN-1 (ATG6), ATG12-ATG5, and LC3 (ATG8) triggering autophagosome formation. Additionally, cleavage of LC3-I to LC3-II is a standard marker of autophagy (Codogno and Meijer, 2005[10]; Levine et al., 2008[24]). Currently, the most effective cytotoxic drug used for glioblastoma treatment is TMZ, which induces autophagic cell death (Kanzawa et al., 2004[22]). In the present study, we determined that the autophagy signaling pathway was activated by both TMZ and EP treatment, especially in U87 cells. Furthermore, both autophagy sensor staining and determination of LC3-I conversion to LC3-II by Western blot clearly supported stronger induction of autophagy in U87 than U87-R cells. These results parallel the findings of Jalota and colleagues (2018[19]), who reported that co-treatment of many alkylating agents causes resistance and supresses mechanisms of autophagy and apoptosis in U87 MG, GBM8401, LN229, and A172 cells.

The most important reasons for failure of glioblastoma treatment are local recurrence of cancer cells and the high angiogenic capacity of secondary tumors. Moreover, high tumor hypoxia, uncontrolled cell proliferation, and abnormal tumoral vascularization are all characteristics of GBM and are directly related to poor prognosis (Muz et al., 2015[27]). The role of hypoxia-induced angiogenesis in drug resistance is well known (Rohwer and Cramer, 2011[33]), with increased expression of HIF1-α causing increases in the VEGFR2-mediated VEGF signal and in expression of MMP2, MMP3, and MMP9 (Conway et al., 2001[11]; Carmeliet and Jain, 2011[8]). In tumor organoids and cell lines, it has been reported that hypoxia-induced signal enhancement after chemotherapy treatment activates cell cycle checkpoints and reduces cell proliferation, leading to drug resistance (Vaupel et al., 2001[42]; Das et al., 2008[13]). In addition, HIF-1α directly binds ABC cassette-type proteins, activating intracellular drug detoxification and triggering multi-drug resistance (Abraham et al., 2015[1]; Popescu et al., 2016[32]). Similar to previous studies, we found the expression of angiogenesis-related genes *VEGF*, *HIF-1α*, *MMP3*, and *MMP9* to be significantly higher in U87-R than normal U87 cells, especially at 24 hours after etoposide treatment. 

## Conclusion

Our results indicate that repeated treatment of the U87 MG cell line with a combination of 5-Fu, cisplatin, and paclitaxel induces cross-resistance, causing MDR against other chemotherapeutic agents that are distinctly different in terms of chemical structure and mechanism of action. The stability of this MDR phenotype across five passages without any chemotherapy treatment suggests that it is inherited across generations, probably as a result of a genetic rearrangement. In addition, these cells exhibit elevated gene or protein expression of MDR biomarkers such as MRP1/ABCC1, ABCC2, BRCP/ABCG2, and GST after treatment with TMZ or EP, contributing key evidence that their resistance is due to the MDR mechanism. Our results also indicate that the resistant U87 MG cells, which were subjected to constant low doses of nominally sensitizing cytotoxic agents in the tumor microenvironment, learn to specifically manage oxidative stress, cell cycle, apoptosis, and autophagy processes in a way that ensures their survival in the face of maintenance chemotherapy treatments, a capability not evidenced by non-resistant U87 cells given the same treatments at the same doses. Moreover, resistant cells showed significantly more active HIF1/VEGF/MM9 signals and elevated wound healing capability relative to control cells. 

There are several limitations of this study, such as its being based on an *in vitro* model and not addressing some fundamental questions regarding the chemotherapy agents, such as their capacity to pass the blood-brain barrier, tissue absorption from blood, and ultimate tissue distribution. However, we feel our data do provide important answers regarding causes and consequences of potential side effects of long-term high-dose combination therapies.

Taken together, all these findings support our hypothesis that the combined administration of high doses of classical antineoplastic agents intended to sensitize tumors to immunotherapy or radiotherapy may ultimately trigger multi-drug resistance. Therefore, we recommend that when deciding on the use of high-dose antineoplastic combination therapy to prolong survival of patients having persistent and resistant GBM tumors, the long-term effects of this treatment on cross-resistance and MDR phenotype should be analyzed in primary cell cultures obtained from patients. 

## Declaration

### Authors' contributions

Oğuzhan Doğanlar: Conceptualization, funding acquisition, project administration, data curation, formal analysis, investigation, writing - review & editing. Zeynep Banu Doğanlar: Project administration, data curation, formal analysis, investigation. Suat Erdoğan: Project administration. Emre Delen: Investigation

### Funding information

This study was funded by the Trakya University Scientific Research Fund (TUBAP 2017/51).

### Compliance with ethical standards 

The authors declare that there is no conflict of interest. This article does not contain any studies with the use of humans and animals as study objects.

## Supplementary Material

Supplementary data

## Figures and Tables

**Table 1:**
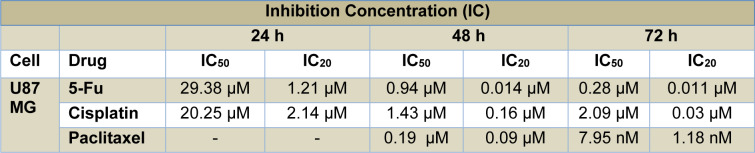
Inhibition concentrations (IC_50_ and IC_20_) in U87 MG cells treated for 24, 48, and 72 h with 5-fluorouracil, cisplatin, and paclitaxel at nine different doses (obtained by serial dilution from stock concentrations of 1000 µM, 200 µM, and 500 nM, respectively). Values were calculated from MTT data using the probit analysis function of SPSS20.

**Table 2:**
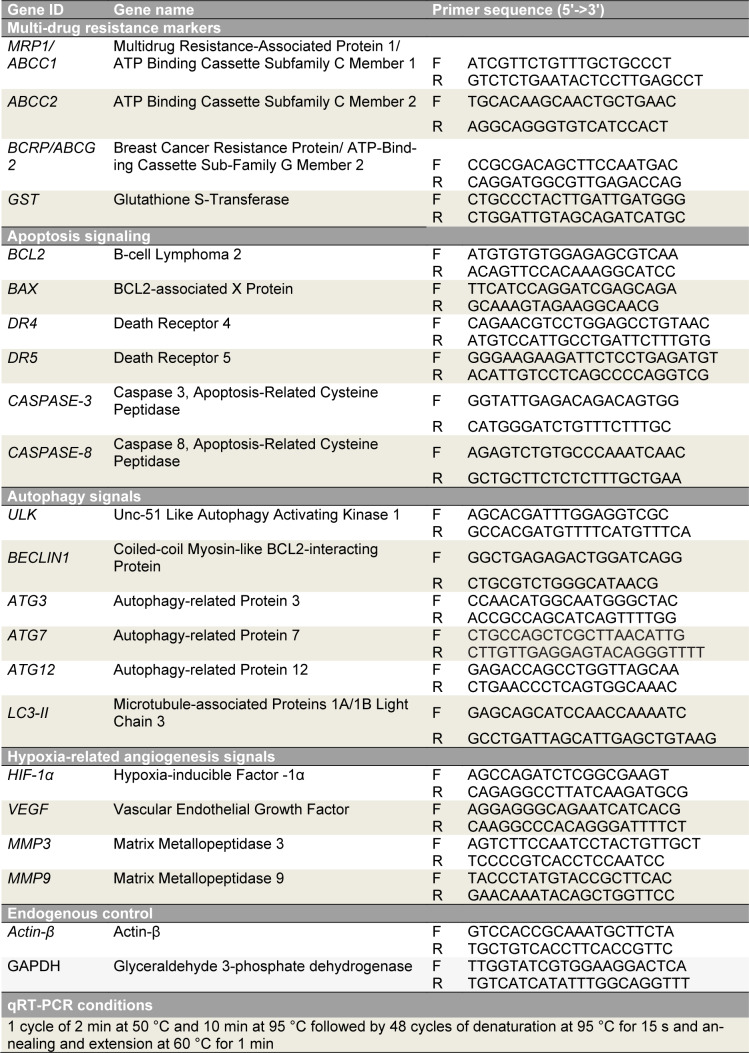
Genes, primer sequences, and PCR conditions used in qRT-PCR assays

**Figure 1:**
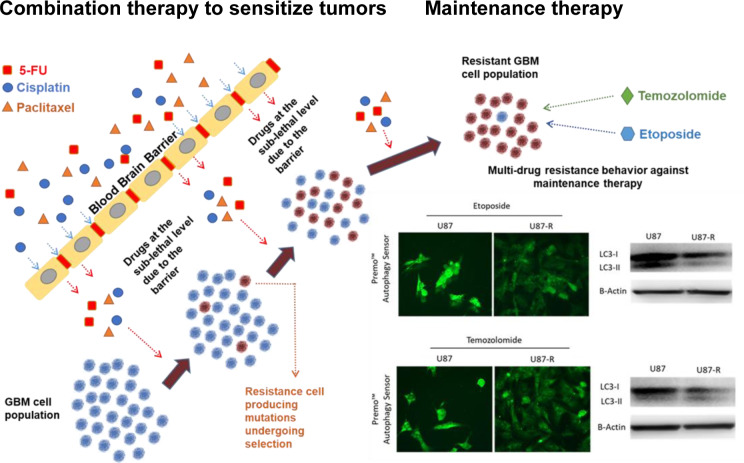
Graphical abstract
